# Association between red cell distribution width—albumin ratio and all-cause mortality in intensive care unit patients with heart failure

**DOI:** 10.3389/fcvm.2025.1410339

**Published:** 2025-01-20

**Authors:** Ni Li, Junling Li, Kai Wang

**Affiliations:** ^1^ Department of Cardiology, Bishan Hospital, Chongqing University of Chinese Medicine, Chongqing, China; ^2^ Department of Cardiology, The Second Affiliated Hospital of Chongqing Medical University, Chongqing, China

**Keywords:** heart failure, intensive care unit, inflammation, prognosis, red cell distribution width—albumin ratio

## Abstract

**Aim:**

The association between red cell distribution width—albumin ratio (RAR) and the risk of all-cause mortality in intensive care unit (ICU) patients with heart failure remains uncertain. This study aimed to investigate this association.

**Methods:**

Clinical data from MIMIC-Ⅳ (version 2.2) database was utilized for the analysis of ICU patients with heart failure. Patients were categorized into quartiles (Q1–Q4) based on RAR levels. Kaplan-Meier survival analysis and multivariate adjusted Cox regression models were employed to assess the association between RAR levels and mortality outcomes within 1 year. Subgroup analysis was used to evaluate the prognostic impact of RAR across diverse populations. Restricted cubic spline curves and threshold effect analysis were utilized to quantify the dose-response relationship between RAR levels and mortality. The time-concordance index curve was carried out to explore the additional prognostic value of RAR on mortality over the existing scoring systems, Serial Organ Failure Assessment (SOFA) and Acute Physiology and Chronic Health Evaluation Ⅱ (APACHE Ⅱ).

**Results:**

The analysis encompassed a cohort of 4,506 patients, with Kaplan-Meier curves indicating that individuals with higher RAR levels exhibited an elevated risk of all-cause mortality (*p* < 0.001). Multivariate adjusted Cox regression and subgroup analysis demonstrated that individuals in Q2 [hazard ratio (HR) 1.15, 95%CI 0.98–1.34], Q3 (HR 1.65, 95%CI 1.39–1.96) and Q4 (HR 2.16, 95%CI 1.74–2.68) had an increased risk of mortality compared to individuals in Q1 (*p* for trend < 0.001), and this relationship was consistently observed across most subgroups, except for different ages. Subsequent analysis revealed that the inclusion of RAR significantly improved the prognostic value on the basis of SOFA and APACHE Ⅱ, and the concordance index increased from 0.636 to 0.658 for SOFA, from 0.682 to 0.695 for APACHE Ⅱ (*p* < 0.001 for both).

**Conclusion:**

The study found that high level of RAR was independently associated with an increased risk of 1-year all-cause mortality in ICU patients with heart failure, with a stronger effect in young and middle-aged patients and a threshold effect, which could potentially serve as an early warning indicator for high-risk populations.

## 1 Introduction

Despite notable advancements in heart failure treatment in recent years, there has not been a significant decline in mortality rates (1). The global prevalence of heart failure exceeds 64 million individuals, with a rising incidence linked to an aging population (2). In the United States, approximately 10%–51% of hospitalized patients with heart failure necessitate intensive care unit (ICU) admission, with a notably elevated risk of mortality (3). Precise evaluation of the condition and its associated risks, along with proactive interventions could enhance the prognosis of heart disease (4). Consequently, patient risk stratification emerged as a crucial strategy in the management of ICU and heart failure. The utilization of the Serial Organ Failure Assessment (SOFA) and Acute Physiology and Chronic Health Evaluation Ⅱ (APACHE Ⅱ) scores for evaluating disease severity and mortality risk is widespread practice in ICU (5), yet discrepancies in scores among certain patients complicate the clinical decision-making. Therefore, the development of additional prognostic stratification tools is necessary to aid these scores. Furthermore, given the variability in healthcare standards across regions, the identification of straightforward and readily available prognostic markers for ICU heart failure is imperative.

In recent years, there has been a growing recognition for inflammatory mechanisms as pivotal contributors in the aggravation and progression of heart failure (6). Moreover, studies emphasized the substantial impact of prolonged inflammation, specially accumulation of inflammation, on the progression of heart failure (7). The red cell distribution width (RDW), a frequently utilized and easily accessible hematology parameter, indicates the heterogeneity of red cell size. Studies have demonstrated that systemic inflammatory conditions can impede maturation and affect erythrocyte morphology, leading to heightened heterogeneity in erythrocyte volume (RDW) (8). Therefore, RDW could serve as a promising biomarker for inflammation and oxidative stress, and has been linked to overall mortality in critically ill individuals. Serum albumin, a negative acute phase protein primarily synthesized by the liver, serves as a marker for nutritional status and inflammatory exudation (9). Based on recent data, serum albumin levels are associated with clinical outcomes in critical illness (10, 11). Furthermore, the RDW to albumin ratio (RAR), a biomarker derived from RDW and albumin, has garnered considerable interest for its reliability and accessibility. Previous studies have indicated that RAR may be an important prognostic tool in critically ill patients with sepsis (12), acute pancreatitis (13), acute myocardial infarction (14). However, these researches have primarily examined the link between RAR and acute illnesses. It is crucial to evaluate the long-term outcomes in individuals with heart failure, due to the chronic and acutely deteriorative nature of heart failure. Given the substantial relationship between inflammation and long-term adverse event risk in patients with heart failure (15, 16), this study will focus on RAR, a novel marker of inflammation, and poor medium- and long-term outcomes in ICU patients with heart failure.

In this study, we aimed to investigate the association between RAR and the 1-year risk of mortality in ICU patients with heart failure, as well as to investigate the potential improvement of SOFA and APACHE Ⅱ scores by RAR.

## 2 Methods

### 2.1 Study database

The study population was sourced from the Medical Information Mart for Intensive Care IV database (MIMIC-Ⅳ, version 2.2), a comprehensive single-center database comprising data from over 190,000 ICU patients from 2008 to 2019. The human participant procedures in this study were conducted in accordance with ethical standards outlined by institutional and/or national research councils, as well as the 1964 Helsinki declaration and its subsequent revisions or equivalent ethical guidelines. The study conducted secondary analysis of de-identified patient data from a specified dataset, thereby exempting the need for obtaining informed consent from participants. Additionally, ethical review was considered unnecessary due to the public availability of the data and the inability to identify individuals directly or indirectly.

### 2.2 Study population

**Figure 1:**
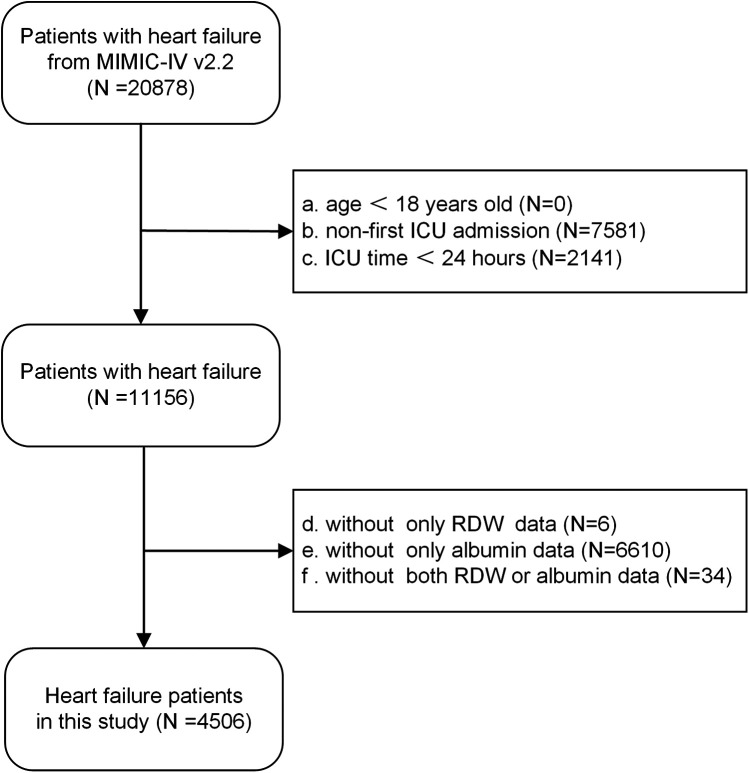
Flow diagram of the number of study participants.

Strict inclusion criteria were applied. Inclusion criteria: all patients diagnosed with congestive heart failure, identified through the use of International Classification of Diseases, Ninth and Tenth Revision (ICD-9 and ICD-10) codes (Supplementary Table 1). Exclusion criteria: (a) under 18 years of age, (b) not admitted to the ICU for the first time, (c) with an ICU stay of less than 24 h, (d) without red cell distribution width or albumin data. Ultimately, the study enrolled a total of 4,506 patients, as illustrated in the flow chart (Figure 1).

### 2.3 Variables acquisition and definition

Clinical data were initially collected within 24 h after admission to ICU, encompassing the demographic characteristics (age, gender), vital signs [heart rate, respiratory rate, saturation of peripheral oxygen (SpO2), blood pressure], laboratory tests [white blood cell count (WBC), neutrophils, red blood cell count (RBC), hemoglobin, red cell distribution width (RDW), platelets, total protein, albumin, aspartate aminotransferase (AST), alanine aminotransferase (ALT), alkaline phosphatase (ALP), total bilirubin, urea nitrogen (BUN), creatinine, albuminuria, sodium, potassium, calcium, chloride, potassium], comorbidities [diabetes, hypertension, chronic obstructive pulmonary disease (COPD), chronic kidney disease (CKD), sepsis], disease severity scores (SOFA and APACHE Ⅱ), medication details [dopamine, dobutamine, milrinone, epinephrine, norepinephrine, adrenaline, vasopressin], mechanical ventilation usage, continuous renal replacement therapy (CRRT), left ventricular ejection fraction (LVEF) and follow-up data. The database provided information on mortality within 1 year for all patients. Due to the prognostic value of malnutrition in patients with heart failure, the prognostic nutritional index (PNI) was calculated as PNI=albumin(g/L)+5×lymphocytes(×109/L) (17). The use of inotropes or vasopressors was defined as the requirement for dopamine, dobutamine, milrinone, phenylephrine, norepinephrine, epinephrine, or vasopressin. The calculation RAR was performed using the formula: RAR = red cell distribution width (%)/albumin (g/dl). The primary endpoint was all-cause mortality within 1 year after discharge.

### 2.4 Statistical analysis

First, descriptive statistics were utilized to display the data, with means and standard deviations (SD) applied to normally distributed continuous variables. Categorical variables were summarized through frequencies and percentages. Baseline characteristics were summarized according to the quartiles (Q1, Q2, Q3 and Q4) of RAR. Relevant statistical tests, such as the chi-square test, ANOVA, or Kolmogorov-Smirnov test, were employed as deemed appropriate.

Then, Kaplan-Meier survival analysis and Log-rank test were employed to evaluate the risk of the primary endpoint in patients with varying RAR levels.

Following this, RAR was assessed as a categorical (quartiles, the lowest quartile served as the reference category), ordinal (increase per quartile), and continuous variable (increase per unit) in four distinct Cox regression models to determine the hazard ratio (HR) and 95% confidence interval (CI) for all-cause mortality, as well as conducting trend tests. Based on previous studies and clinical experience, Model 0 was solely incorporated RAR without any covariate adjustment, Model 1 was adjusted for demographic characteristics (age, gender), comorbidities (diabetes, hypertension, CKD, sepsis, PNI), Model 2 was adjusted for the variables in Model 1 plus situation of inflammation and anemia (neutrophils, hemoglobin), dysfunction of liver and kidney (albumin, total bilirubin, BUN, creatinine), use of medications and instrument (inotropes or vasopressors, CRRT, mechanical ventilation), Model 3 was adjusted for the variables in Model 2 plus SOFA and APACHE Ⅱ.

Subsequently, restricted cubic spline analysis was utilized to capture the dose-response relationship between RAR and all-cause mortality, with threshold effect analysis conducted as necessary.

Furthermore, subgroup analysis and interaction tests were conducted using Model 3 to assess the prognostic significance of RAR in various populations. The analysis took into account various demographic and clinical factors, including age, gender, presence of hypertension, diabetes, COPD, CKD, sepsis, as well as utilization of inotropes or vasopressors, CRRT, mechanical ventilation, and SOFA- APACHE Ⅱ matched [(SOFA<7 & APACHE Ⅱ<64) or (SOFA≥7 & APACHE Ⅱ≥64)] or unmatched [(SOFA<7 & APACHE Ⅱ≥64) or (SOFA≥7 & APACHE Ⅱ<64)]) groups.

Additionally, a time-concordance index curve was carried out to explore the additional prognostic value of RAR on mortality. Predictors including RDW, albumin, RDW plus albumin, RAR, SOFA, APACHE Ⅱ, SOFA plus RAR, and APACHE Ⅱ plus RAR were used to generate curves.

Finally, in order to ensure the robustness of the analysis findings, a sensitivity analysis was performed. Risk ratios (RR) for RAR quartiles were obtained using univariate and multivariate modified Poisson regression, with all-cause mortality within 1 year as the outcome. All statistical analyses were carried out using R software (version 4.3.1), with a significance level of *p* < 0.05.

## 3 Results

### 3.1 Comparisons of the characteristics between groups

The study included 4,506 patients from MIMIC-Ⅳ. The Table 1 presented the baseline characteristics of all participants, categorized by quartiles of RAR (Q1: 2.50–3.96, Q2: 3.97–4.67, Q3: 4.68–5.78, Q4: 5.79–18.60). Among these patients, those with higher RAR exhibited increased mean levels of heart rates (88.98, 89.20, 91.31 vs. 92.65/min for Q1–Q4), RDW (13.79, 14.92, 16.15 vs. 18.15%), neutrophils (9.66, 10.10, 10.39 vs. 11.33 ×10^9^/L), liver damage (81.00, 85.00, 92.00 vs. 102.00 U/L of ALP; 0.60, 0.60, 0.70 vs. 0.80 mg/dl of total bilirubin) and kidney damage (24.00, 29.00, 34.00 vs. 34.00 mg/dl of BUN; 1.20, 1.30, 1.50 vs. 1.40 mg/dl of creatinine), higher proportion of CKD (14.9, 24.1, 25.8% vs. 23.3%), sepsis (55.8, 62.2, 68.4 vs. 77.8%), inotropes or vasopressors usage (29.6, 36.1, 43.5 vs. 50.3%), CRRT (3.9, 7.0, 8.3 vs. 11.1) and ventilation (36.1, 37.6, 42.8 vs. 43.4%), elevated mean SOFA (5.32, 6.08, 7.09 vs. 7.93) and APACHE Ⅱ (58.30, 63.32, 69.75 vs. 77.22), and decreased mean levels of albumin (3.90, 3.46, 3.13 vs. 2.57 g/dl), lymphocytes ( 1.16, 1.05, 0.99 vs. 0.94), PNI (44.95, 40.10, 36.51 vs. 30.81) (*p* < 0.001 for all). Endpoint events occurred in 43.1% (1,944/4,506) of patients. Patients with higher RAR had higher all-cause mortality with 28.2%, 34.7%, 49.1% and 60.5% (*p* < 0.001). Patients with mortality had higher levels of RAR than those with survival (5.55 vs. 4.71) (Supplementary Figure 1).

**Table 1:** Baseline characteristics of study participants according to quartiles of red cell distribution width—albumin ratio.

	Overall (2.50–18.60) (*n* = 4,506)	Q1 (2.50–3.96) (*n* = 1,126)	Q2 (3.97–4.67) (*n* = 1,127)	Q3 (4.68–5.78, Q4) (*n* = 1,126)	Q4 (5.79–18.60) (*n* = 1,127)	*p* value
Age (years)	72.08 (14.38)	71.16 (14.92)	72.96 (14.38)	72.69 (13.72)	71.51 (14.43)	0.005
Male, *n* (%)	2,537 (56.3)	702 (62.3)	616 (54.7)	597 (53.0)	622 (55.2)	<0.001
Diabetes, *n* (%)	1,836 (40.7)	430 (38.2)	459 (40.7)	482 (42.8)	465 (41.3)	0.161
Hypertension, *n* (%)	3,412 (75.7)	889 (79.0)	880 (78.1)	853 (75.8)	790 (70.1)	<0.001
COPD, *n* (%)	913 (20.3)	195 (17.3)	225 (20.0)	272 (24.2)	221 (19.6)	0.001
CKD, *n* (%)	993 (22.0%)	168 (14.9%)	272 (24.1%)	290 (25.8%)	263 (23.3%)	<0.001
Sepsis, *n* (%)	2,976 (66.0%)	628 (55.8%)	701 (62.2%)	770 (68.4%)	877 (77.8%)	<0.001
Heart rate (/min)	90.53 (21.41)	88.98 (20.36)	89.20 (20.13)	91.31 (22.44)	92.65 (22.43)	<0.001
Resp rate (/min)	20.94 (6.33)	20.59 (6.10)	21.03 (6.24)	21.10 (6.28)	21.02 (6.69)	0.202
SpO2 (%)	97.00 (94.00, 100.00)	97.00 (95.00, 99.00)	97.00 (94.00, 100.00)	97.00 (95.00, 100.00)	97.00 (94.00, 100.00)	0.105
SBP (mmHg)	121.56 (25.77)	128.47 (25.82)	123.65 (25.95)	119.43 (25.25)	114.68 (24.00)	<0.001
WBC (10^9^/L)	11.00 (7.90, 15.70)	10.70 (7.90, 14.68)	11.00 (8.20, 15.10)	11.10 (7.53, 15.90)	11.50 (7.60, 17.15)	0.104
Hemoglobin (g/dl)	10.88 (2.48)	12.55 (2.06)	11.16 (2.26)	10.28 (2.26)	9.51 (2.27)	<0.001
Platelets (10^9^/L)	204.00 (149.00, 277.00)	209.00 (166.00, 269.75)	211.00 (161.00, 276.00)	203.00 (137.00, 277.00)	193.00 (125.00, 293.00)	<0.001
RDW (%)	15.75 (2.56)	13.79 (1.01)	14.92 (1.34)	16.15 (1.88)	18.15 (3.06)	<0.001
Neutrophils (10^9^/L)	10.37 (6.75)	9.66 (5.37)	10.10 (6.10)	10.39 (6.91)	11.33 (8.17)	<0.001
Lymphocytes (10^9^/L)	1.03 (0.63, 1.60)	1.16 (0.74, 1.74)	1.05 (0.65, 1.68)	0.99 (0.60, 1.51)	0.94 (0.56, 1.47)	<0.001
PNI	38.75 (33.60, 43.80)	44.95 (41.60, 49.14)	40.10 (36.97, 43.90)	36.51 (33.31, 40.09)	30.81 (26.90, 35.11)	<0.001
Total protein (g/dl)	5.58 (1.04)	6.16 (0.86)	5.75 (0.92)	5.47 (1.00)	4.94 (0.98)	<0.001
Albumin (g/dl)	3.26 (0.62)	3.90 (0.35)	3.46 (0.32)	3.13 (0.36)	2.57 (0.50)	<0.001
ALT (U/L)	26.00 (16.00, 54.00)	27.00 (17.25, 52.00)	27.00 (17.00, 57.00)	26.50 (16.00, 55.00)	25.00 (15.00, 54.00)	0.014
AST (U/L)	39.00 (24.00, 84.00)	38.00 (24.00, 83.00)	39.00 (24.00, 81.00)	41.00 (24.00, 83.00)	40.00 (24.00, 87.00)	0.982
ALP (U/L)	89.00 (66.00, 128.00)	81.00 (64.00, 105.75)	85.00 (65.50, 119.00)	92.00 (66.00, 137.00)	102.00 (71.00, 155.00)	<0.001
Total bilirubin (mg/dl)	0.70 (0.40, 1.20)	0.60 (0.40, 0.90)	0.60 (0.40, 1.00)	0.70 (0.40, 1.20)	0.80 (0.40, 1.70)	<0.001
BUN (mg/dl)	30.00 (19.00, 48.00)	24.00 (17.00, 36.75)	29.00 (19.00, 47.00)	34.00 (21.00, 55.00)	34.00 (21.00, 55.00)	<0.001
Creatinine (mg/dl)	1.40 (0.90, 2.20)	1.20 (0.90, 1.70)	1.30 (0.90, 2.10)	1.50 (1.00, 2.40)	1.40 (1.00, 2.50)	<0.001
Albuminuria (mg/dl)	77.00 (30.00, 77.00)	77.00 (30.00, 77.00)	77.00 (30.00, 77.00)	77.00 (30.00, 100.00)	77.00 (30.00, 77.00)	0.177
Glucose (mg/dl)	136.00 (108.00, 184.00)	146.00 (115.00, 197.00)	139.00 (113.00, 189.00)	134.50 (107.00, 185.00)	125.00 (100.00, 169.50)	<0.001
LVEF (%)	46.44 (47.15)	43.94 (15.57)	48.58 (90.47)	46.42 (15.21)	46.82 (15.00)	0.134
Inotropes/vasopressors, *n* (%)	1,797 (39.9)	333 (29.6)	407 (36.1)	490 (43.5)	567 (50.3)	<0.001
CRRT, *n* (%)	342 (7.6)	44 (3.9)	79 (7.0)	94 (8.3)	125 (11.1)	<0.001
Ventilation, *n* (%)	1,801 (40.0)	406 (36.1)	424 (37.6)	482 (42.8)	489 (43.4)	<0.001
SOFA	6.60 (4.00)	5.32 (3.56)	6.08 (3.64)	7.09 (4.00)	7.93 (4.26)	<0.001
APACHE Ⅱ	67.15 (24.64)	58.30 (21.29)	63.32 (21.70)	69.75 (24.49)	77.22 (26.53)	<0.001
Mortality, *n* (%)	1,944 (43.1)	318 (28.2)	391 (34.7)	553 (49.1)	682 (60.5)	<0.001

COPD, chronic obstructive pulmonary disease; CKD, chronic kidney disease; SBP, systolic blood pressure; SpO2, saturation of peripheral oxygen; WBC, white blood cell count; RDW, red cell distribution width; PNI, prognostic nutritional index; ALT, alanine aminotransferase; AST, aspartate aminotransferase; ALP, alkaline phosphatase; BUN, blood urea nitrogen; LVEF, left ventricular ejection fraction; CRRT, continuous renal replacement therapy; SOFA, sequential organ failure assessment; APACHE Ⅱ, acute physiology and chronic health evaluation Ⅱ.

In addition, as described in Supplementary Table 2, patients with RAR levels less than 5.25 had higher levels of heart rate (92.71 vs. 89.36/min), WBC (11.60 vs. 10.80 × 10^9^/L), RDW (17.20 vs. 14.50), neutrophils (9.35 vs. 8.59 × 10^9^/L), ALP (98.00 vs. 85.00 U/L), BUN (35.00 vs. 27.00 mg/dl), creatinine (1.50 vs. 1.30 mg/dl), SOFA (7.84 vs. 5.94), APACHE Ⅱ (75.91 vs. 62.43), a higher proportion of CKD (24.6% vs. 20.7%), sepsis (75.6% vs. 60.9%), inotropes or vasopressors (48.7% vs. 35.1%), CRRT (10.5% vs. 6.0%), mechanical ventilation (44.1% vs. 37.7%), and lower mean levels of SBP (115.54 vs. 124.79 mmHg), hemoglobin (9.67 vs. 11.52 g/dl) than patients with RAR levels greater than 8.04.

### 3.2 Kaplan-Meier survival curve

**Figure 2:**
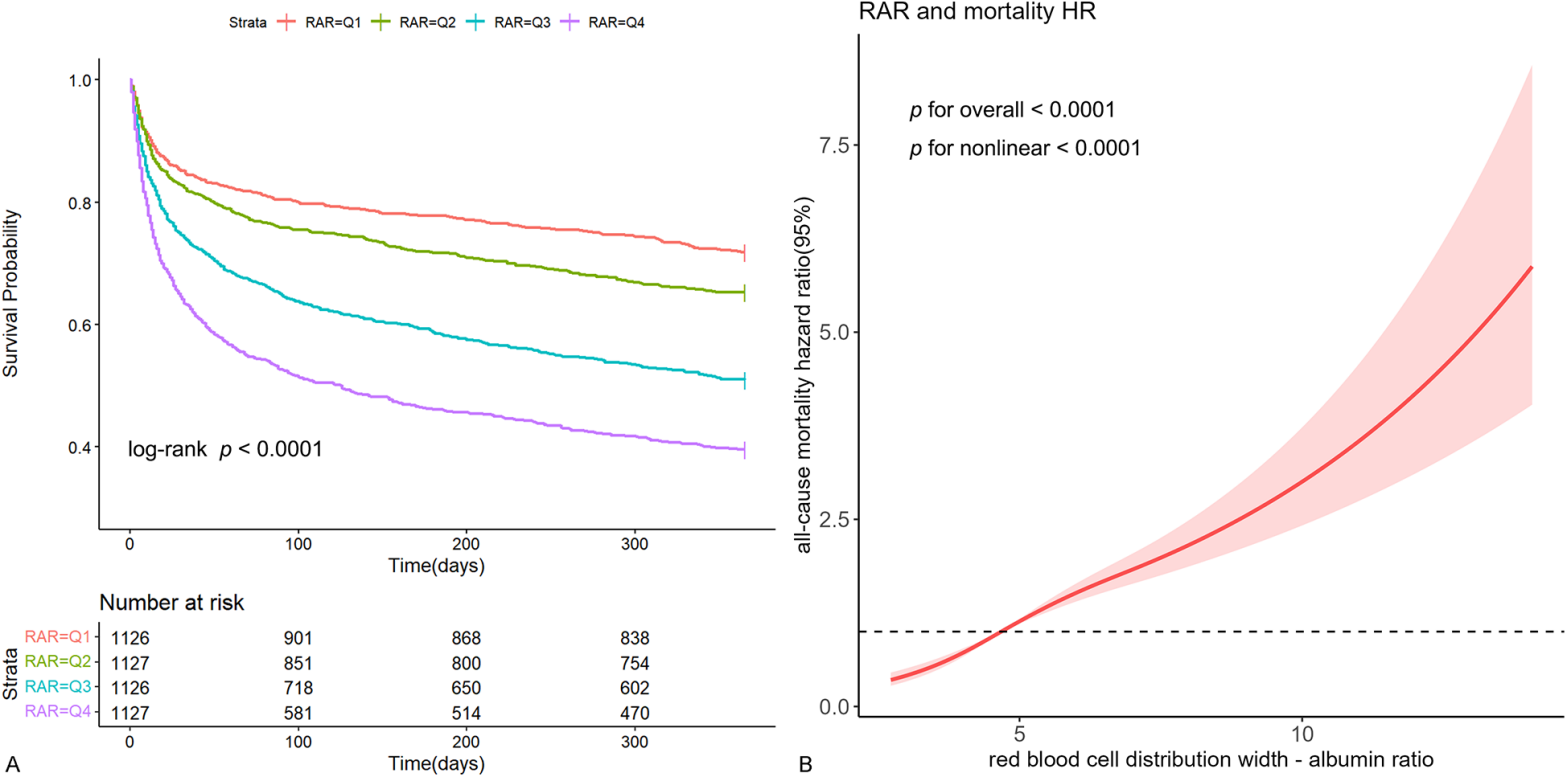
Kaplan-Meier survival curve and restricted cubic spline curve. Cumulative incidence of all-cause mortality according to RAR quartiles **(A)** and cubic spline model of the association between RAR and risk of all-cause mortality in all patients adjusted with age, gender, diabetes, hypertension, CKD, sepsis, PNI score, neutrophils, hemoglobin, albumin, total bilirubin, BUN, creatinine, inotropes or vasopressors, LVEF, CRRT, ventilation, SOFA and APACHE Ⅱ **(B).**

Kaplan-Meier survival curve analysis was used to assess the cumulative survival rate. Figure 2A illustrated the 1-year cumulative survival rates for the four groups (Q1, Q2, Q3, and Q4) categorized by RAR quartiles. Individuals with higher RAR levels demonstrated a notably worse prognosis in comparison to those with lower RAR levels.

### 3.3 Hazard ratios for all-cause mortality

Upon adjusting for age, gender, diabetes, hypertension, CKD, sepsis, PNI score, neutrophils, hemoglobin, albumin, total bilirubin, BUN, creatinine, inotropes or vasopressors, LVEF, CRRT, ventilation, SOFA and APACHE Ⅱ (Model 3), the presence of elevated RAR was independently associated with high all-cause mortality (HR: 1.15, 95% CI: 0.98–1.34 for Q2; HR: 1.65, 95% CI: 1.39–1.96 for Q3; HR: 2.16, 95% CI: 1.74–2.68 for Q4 compared with Q1) (*p* for trend < 0.001) (Table 2). For each unit increase in RAR, the risk of all-cause mortality increased by 20% (HR: 1.20, 95% CI: 1.15–1.25) (Table 2).

**Table 2:** Cox models for the association between red cell distribution width—albumin ratio and all-cause mortality.

RAR	Case/Total	Model 0	Model 1	Model 2	Model 3
Quartiles	–	Hazard ratio			
Q1	1,126/4,506	Reference	Reference	Reference	Reference
Q2	1,127/4,506	1.30 (1.10, 1.50)	1.16 (1.00, 1.35)	1.09 (0.93, 1.28)	1.15 (0.98, 1.34)
Q3	1,126/4,506	2.00 (1.80, 2.30)	1.83 (1.59, 2.10)	1.60 (1.35, 1.90)	1.65 (1.39, 1.96)
Q4	1,127/4,506	2.80 (2.50, 3.20)	2.59 (2.26, 2.97)	2.12 (1.72, 2.63)	2.16 (1.74, 2.68)
*p* for trend	–	<0.001	<0.001	<0.001	<0.001
Per quartile increase	–	1.44 (1.38, 1.50)	1.41 (1.35, 1.47)	1.32 (1.23, 1.41)	1.32 (1.23, 1.41)
Per unit increase	–	1.23 (1.21, 1.26)	1.24 (1.21, 1.26)	1.23 (1.18, 1.28)	1.20 (1.15, 1.25)

Model 0: red cell distribution width—albumin ratio without adjust; Model 1: age, gender, diabetes, hypertension, CKD, sepsis, and PNI score were adjusted; Model 2: the variables in Model 1 plus neutrophils, hemoglobin, albumin, total bilirubin, BUN, creatinine, inotropes or vasopressors, CRRT and ventilation were adjusted; Model 3: the variables in Model 2 plus SOFA and APACHE Ⅱ were adjusted.

### 3.4 Restricted cubic spline and threshold effect analysis

In the fully adjusted restricted cubic spline model, a nonlinear association between RAR and the risk of all-cause mortality was observed (*p* for nonlinear < 0.001) (Figure 2B). Additionally, utilizing a two-segment COX regression model and a recursive algorithm, the inflection point of the association between RAR levels and the risk of all-cause mortality was identified as 5.25. Below this threshold, each incremental unit in RAR was linked to a 64% increase in the risk of all-cause mortality (HR: 1.64, 95% CI: 1.47–1.84). When RAR level exceeded 5.25, each incremental unit in RAR was linked to a 19% increase in the risk of all-cause mortality (HR: 1.19, 95% CI: 1.14–1.24). The results of likelihood ratio tests indicated that the two-segment COX regression model provided a more optimal fit for investigating the association between RAR and the risk of all-cause mortality in comparison to the single-line COX regression model (likelihood ratio test *p* < 0.001) (Supplementary Table 3).

### 3.5 Subgroup analysis

**Figure 3:**
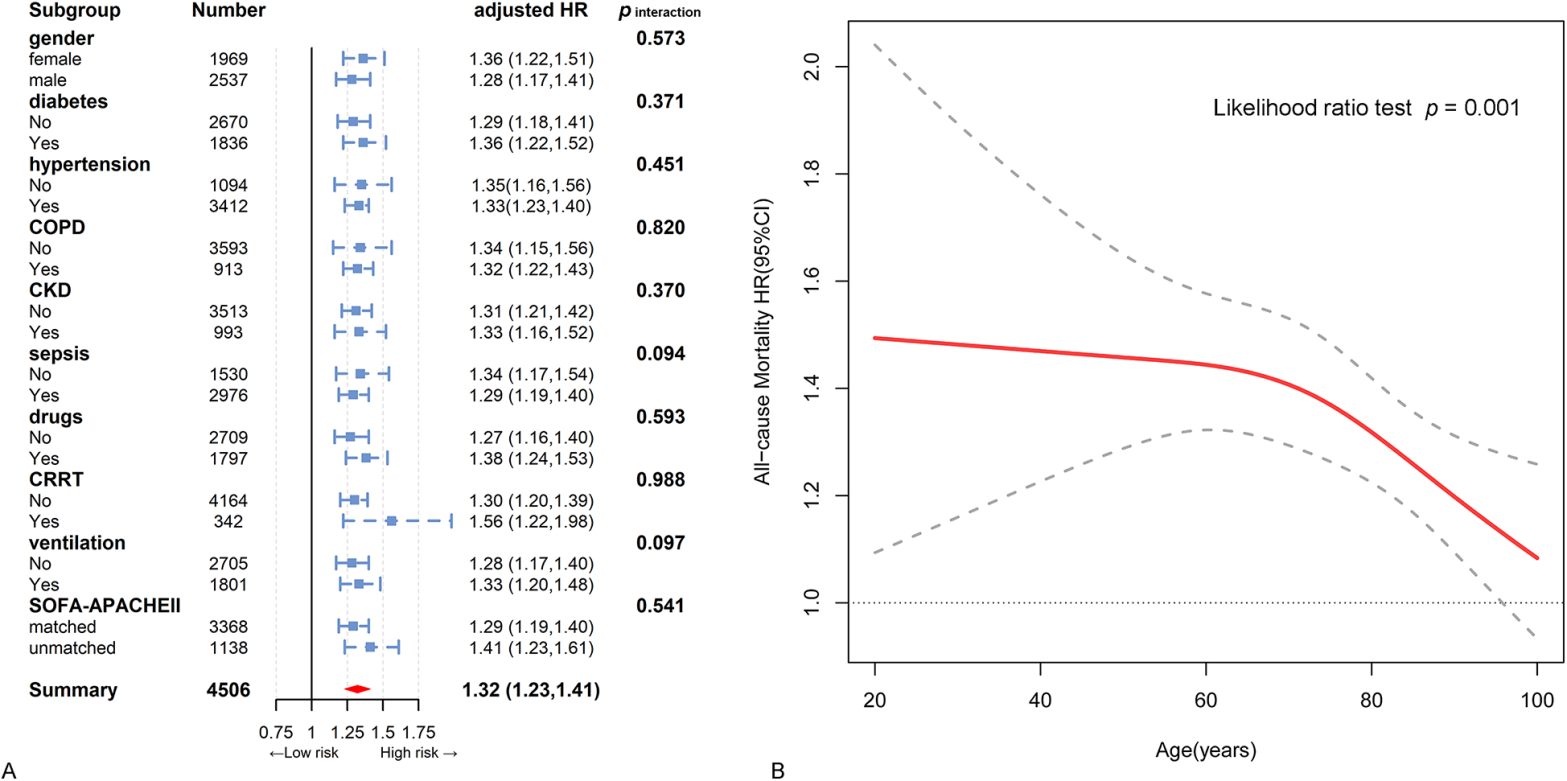
Hazard ratios and error bars delineating 95% confidence intervals from model 3 by subgroups. **(A)** Subgroup analysis by gender, diabetes, hypertension, chronic obstructive pulmonary disease, chronic kidney disease, sepsis, inotropes or vasopressors, continuous renal replacement therapy region, ventilation, and SOFA- APACHE Ⅱ matched. **(B)** The interactive restricted cubic spline curves for subgroup analysis in various levels of age.

Subgroup analysis was conducted with stratification to explore the relationship between RAR and all-cause mortality, along with an interaction analysis (Figure 3). This observed positive relationship was consistent across most subgroups, including gender, diabetes, hypertension, COPD, CKD, sepsis, inotropes or vasopressors, CRRT, ventilation, and matched or unmatched SOFA and APACHE Ⅱ scores (*p* for interaction > 0.05 for all) (Figure 3A). Dissimilarly, the association was stronger in relatively younger patients than in older patients (Figure 3B).

### 3.6 Cut-off value and added power of RAR

Using function of *surv_cutpoint* in *survminer* R package, we found an optimal cut-off value of 4.81 for RAR to assess the risk of all-cause mortality in this population (Supplementary Figure 2).

**Figure 4:**
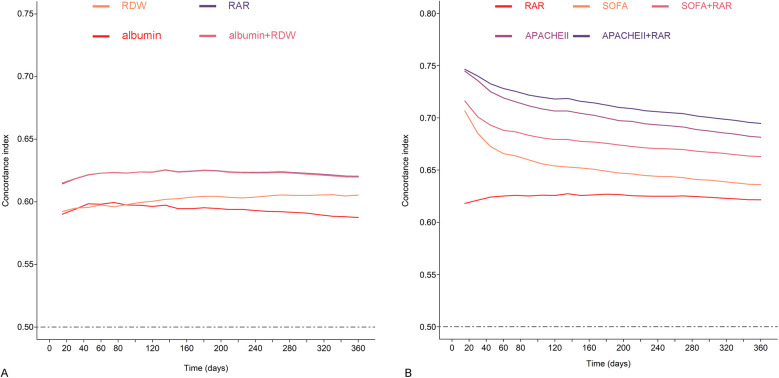
Concordance index curves of all-cause mortality during the follow-up period. **(A)** Concordance index predicted by RDW, albumin, RDW plus albumin, and RAR. **(B)** Concordance index predicted by RAR, SOFA, APACHE II, SOFA plus RAR, APACHE II plus RAR.

The findings from the analysis of time-concordance index curve indicated more advanced risk warning values of RAR compared to albumin or RDW, and equivalent value compared to the combination of albumin and RDW (Figure 4A). Subsequent analysis revealed that the inclusion of RAR significantly improved the prognostic value on the basis of SOFA and APACHE Ⅱ, and the concordance index increased from 0.636 to 0.658 for SOFA, from 0.682 to 0.695 for APACHE Ⅱ (*p* < 0.001 for both) (Figure 4B; Supplementary Table 4).

### 3.7 Sensitivity analysis

Modified Poisson regression was used as sensitivity analysis. Regression results demonstrated that RAR quartiles remained both positively and similarly associated with the risk of all-cause mortality (adjusted RR: 1.24, 95% CI: 1.18–1.30, for per quartiles increase; adjusted RR: 1.12, 95% CI: 1.09–1.15, for per unit increase) (Supplementary Table 5).

## 4 Discussion

This study comprehensively investigated the independent association between RAR and all-cause mortality of ICU patients with heart failure, and affirmed its substantial incremental value over SOFA and APACHE Ⅱ.

RAR, identified as a novel and stable inflammatory marker, has been demonstrated to be closely associated with the prognosis of critically ill patients. Xu et al. discovered a significant association between RAR and clinical outcomes in ICU patients with sepsis (12). The higher RAR levels, the higher in-hospital and 90-day mortality. Another retrospective study further confirmed a strong connection between elevated RAR levels and an increased risk of acute kidney injury in ICU-admitted sepsis patients (18). Seo et al.'s investigation revealed a significant relationship between RAR levels on the first day post-operation in burn patients and ICU hospitalization rate, ICU duration, and 90-day mortality rate, independent of total burn body surface area (19). Similar findings were also found in patients with myocardial infarction and acute pancreatitis (13, 14). Building on these findings, our study aimed to investigate the value of RAR in assessing the risk of all-cause mortality in heart failure patients necessitating ICU care. Through manifold statistical methods, including multivariate adjustments, trend tests, curve-fitting, and subgroup analysis, our findings consistently confirmed that RAR was strongly associated with the risk of all-cause mortality within 1 year for these patients (Table 2; Figures 2A, 3A; Supplementary Figure 2). Additionally, a sensitivity analysis treating all-cause mortality as a binary outcome further supported these findings, which bolstered the reliability of our results (Supplementary Table 5). Moreover, it is interesting to note that our study found that RAR surpassed RDW or albumin in predicting risk of all-cause mortality, while being almost completely equivalent to the combination of RDW and albumin (Figure 4A). This suggests that RAR not only retains all the prognostic information of original variables, but also offers a convenient and efficient way for clinicians to integrate the two variables. These findings may potentially encourage wider adoption and utilization of RAR among physicians.

Our study, using data from the updated MIMIC-Ⅳ database (version 2.2), included a larger sample size of patients with heart failure. More advanced than a recent study analyzed in MIMIC Ⅲ (version 1.4) (20), our findings indicated a positive but nonlinear association between RAR and the risk of mortality in ICU patients with heart failure, with a certain threshold effect (Figure 2B). Further threshold effect analysis determined the inflection point of RAR to be 5.25 (Supplementary Table 3). When RAR was less than 5.25, there was a 64.0% increase in the risk of mortality for each unit increase in RAR, while RAR level was greater than 5.25, there was a 19.0% increase in the risk of mortality for each incremental unit in RAR. As we described, patients with RAR below 5.25 had higher levels of heart rate, RDW, neutrophils, ALP, BUN, creatinine, SOFA, APACHE Ⅱ, higher proportion of CKD, sepsis, inotropes or vasopressors, CRRT, mechanical ventilation, and lower mean levels of SBP, hemoglobin. However, these parameters are strongly associated with the risk of all-cause mortality in heart failure patients. When RAR surpassed 5.25, the levels of these risk factors was elevated, resulting in a diminished effect of RAR. Conversely, when RAR fell below 5.25, the effect of RAR was less affected by these risk factors, leading to a stronger prognostic effect of RAR. This observation may elucidate the non-linear relationship between RAR and the risk of all-cause mortality. Another important difference from that research, our study identified that RAR had a pronounced prognostic effect on younger individuals compared to older individuals (Figure 3B). This finding aligns with the results of Hong et al., who focused on the prognostic value of RAR in diabetic foot ulcer patients and concluded that RAR was a more reliable predictor of mortality in younger and middle-aged individuals (21). It is widely acknowledged that older critically ill patients have a heightened risk of mortality. In contrast, our study revealed that RAR was more effective in providing warnings for younger patients, suggesting its potential utility in risk stratification and identification of high-risk groups among this demographic. Therefore, to a certain extent, our findings have a very important role in expanding the above research. These findings more accurately delineate the dose-response relationship between RAR and risk of all-cause mortality and may be more helpful for clinicians in risk stratification.

The utilization of SOFA and APACHE Ⅱ scoring systems has become integral tools for intensive care physicians in assess the severity of patients (5). Our study, in line with prior studies, suggests that these scores have effective warning value for ICU patients with heart failure. However, it is crucial to acknowledge the inconsistency in the results of these scores in certain patients, with approximately 25% of individuals in our study displaying such discrepancies. More importantly, our findings demonstrate that RAR exhibits consistent prognostic value across populations, regardless of concordant and discordant SOFA and APACHE Ⅱ scores (Figure 3A). Furthermore, based on SOFA and APACHE Ⅱ, the incorporation of RAR may significantly improve the capacity to assess adverse outcomes (Figure 4B; Supplementary Table 4). These results suggest that RAR has the potential to challenge this grey area (fuzzy field) as a powerful supplement to SOFA and APACHE Ⅱ.

In investigations into the relationship between RAR and heart failure, it was observed that inflammation and hypoalbuminemia are significant factors in the advancement of heart failure. Inflammation is a prevalent pathological mechanism in individuals with heart failure (22), while hypoalbuminemia serves as a multifaceted indicator of nutritional status, inflammation, and renal impairment, and is linked to unfavorable outcomes in heart failure patients (23). Moreover, individuals with heart failure frequently present with comorbid conditions such as hypertension and diabetes. The latter can exacerbate heart failure not only directly, but also by exacerbating inflammation and kidney damage. Albuminuria serves as a crucial biomarker for kidney damage (24). Intensive hypotension has been shown to decrease proteinuria and lower the incidence of major cardiovascular adverse events (25). Inhibiting the inflammatory state of the kidney in diabetic nephropathy may enhance renal podocyte function and mitigate kidney damage (26). Additional research has demonstrated that diabetes can stimulate myocardial fibrosis and worsen heart failure through multiple mechanisms, such as impaired energy metabolism, mitochondrial dysfunction, autophagy and endoplasmic reticulum stress, with inflammation exacerbating the aforementioned pathological processes (27). Conversely, enhancing inflammation and mitochondrial function has the potential to ameliorate cardiomyocyte apoptosis and mitigate the occurrence of heart failure (28). Additionally, the inhibition of fibrosis may present a novel therapeutic avenue for individuals suffering from diabetes and heart failure (29). In summary, the utilization of RAR as an inflammatory marker not only enables precise risk assessment for patients, but also serves as a reflection of organ pathology to a certain degree. A comprehensive comprehension of these mechanisms will aid in the advancement and refinement of treatment modalities.

However, this study was not without limitations. First, this investigation represents a *post-hoc* analysis of retrospective cohort. Despite the utilization of multivariate model to manage confounding variables, due to the limitations of the database, certain unidentified and potentially significant risk factors were not taken into account. Specially, acute kidney injury and B-type natriuretic peptide, as two crucial covariates, were not included in this analysis. These may pose a few challenges to the exploration of this study to some extent. Second, it is challenging to determine whether heart failure is the primary cause for admission to ICU in MIMIC-Ⅳ. However, ICU admissions are often caused by critical conditions in multiple organs, including heart failure.

## 5 Conclusion

The study found that high levels of RAR were independently associated with an increased risk of 1-year all-cause mortality in ICU patients with heart failure, with a stronger effect in young and middle-aged patients and a threshold effect, which could potentially serve as an additional reference to facilitate clinical consultation and optimize management decisions in these patients.

## Data Availability

The datasets presented in this study can be found in online repositories. The names of the repository/repositories and accession number(s) can be found below: https://physionet.org/content/mimiciv/2.2/.
