# Supplementary figures and images for: Association between red cell distribution width—albumin ratio and all-cause mortality in intensive care unit patients with heart failure
Source: Front Cardiovasc Med. 2025 Jan 20;12:1410339. doi: 10.3389/fcvm.2025.1410339 (PMC11788307; doi:10.3389/fcvm.2025.1410339)

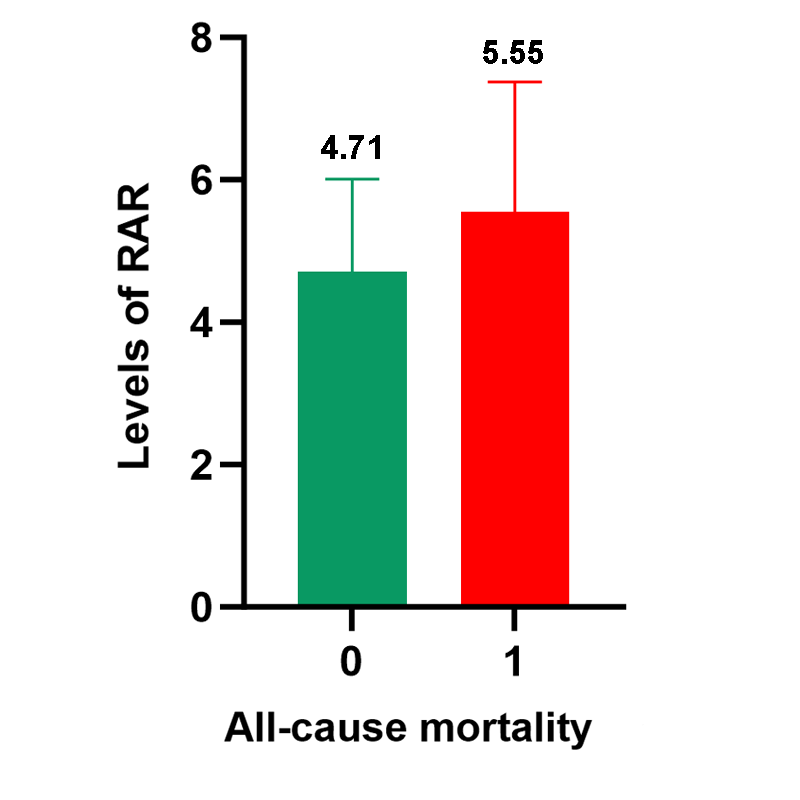

Supplement: Supplementary file 1 [file Image1.tif]

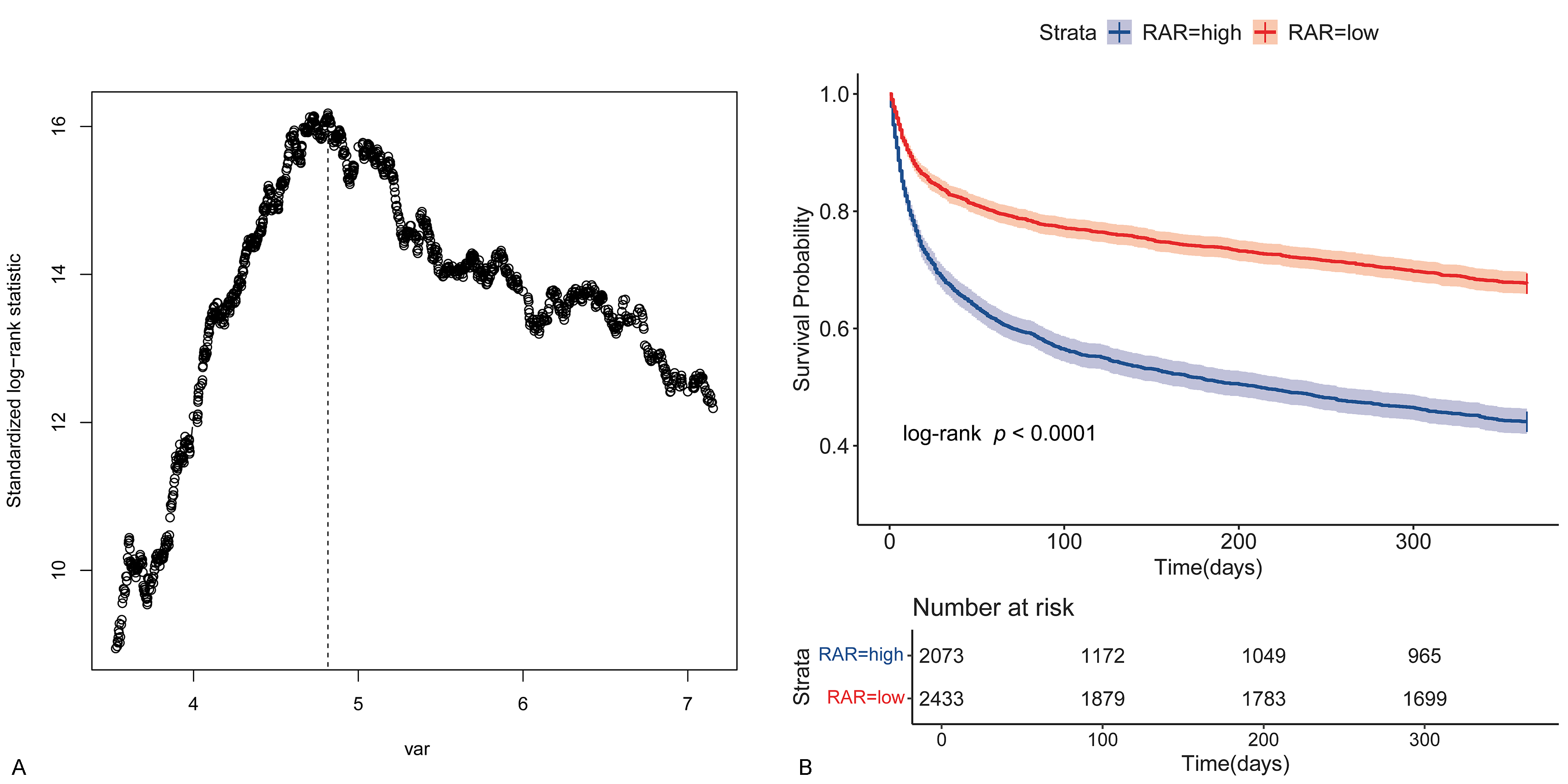

Supplement: Supplementary file 2 [file Image2.tif]
