# Supplementary material for: Association between red cell distribution width—albumin ratio and all-cause mortality in intensive care unit patients with heart failure
Source: Front Cardiovasc Med. 2025 Jan 20;12:1410339. doi: 10.3389/fcvm.2025.1410339 (PMC11788307; doi:10.3389/fcvm.2025.1410339)
Supplement: Supplementary file 3 [file Table1.pdf]

**Supplementary table 1.** ICD-9 and ICD-10 codes used to screen of patients with congestive heart failure.

| ICD-9 | | | ICD-10 |
|---|---|---|---|
| 428 | 4258 | 40411 | I43 |
| 4280 | 4259 | 40413 | I50 |
| 4281 | 4284 | 40491 | I099 |
| 4282 | 42840 | 40493 | I110 |
| 42821 | 42841 | | I130 |
| 42822 | 42842 | | I132 |
| 42823 | 42843 | | I255 |
| 4283 | 4289 | | I420 |
| 42830 | 39891 | | I425 |
| 42831 | 40201 | | I426 |
| 42832 | 40211 | | I427 |
| 4254 | 40291 | | I428 |
| 4255 | 40401 | | I429 |
| 4257 | 40403 | | P290 |

**Supplementary table 2**. Baseline characteristics of study participants according to threshold of RAR from the threshold effect analysis.

| | Overall (n=4506) | RAR < 5.25 (n=2931) | RAR ≥ 5.25 (n=1575) | *p* value |
|---|---|---|---|---|
| age(years) | 72.08 (14.38) | 72.28 (14.36) | 71.71 (14.42) | 0.212 |
| male, n(%) | 2537 (56.3) | 702 (62.3) | 616 (54.7) | <0.001 |
| diabetes, n(%) | 1836 (40.7%) | 1184 (40.4%) | 652 (41.4%) | 0.514 |
| hypertension, n(%) | 3412 (75.7%) | 2288 (78.1%) | 1124 (71.4%) | <0.001 |
| COPD, n(%) | 913 (20.3%) | 583 (19.9%) | 330 (21.0%) | 0.398 |
| CKD, n(%) | 993 (22.0%) | 606 (20.7%) | 387 (24.6%) | 0.003 |
| sepsis, n(%) | 2976 (66.0%) | 1786 (60.9%) | 1190 (75.6%) | <0.001 |
| heart rate(/min) | 90.53 (21.41) | 89.36 (20.78) | 92.71 (22.39) | <0.001 |
| resp rate(/min) | 20.94 (6.33) | 20.87 (6.18) | 21.06 (6.61) | 0.356 |
| SpO2(%) | 97.00 (94.00, 100.00) | 97.00 (95.00, 99.00) | 97.00 (94.00, 100.00) | 0.141 |
| SBP(mmHg) | 121.56 (25.77) | 124.79 (25.92) | 115.54 (24.36) | <0.001 |
| WBC(10^9/L) | 11.00 (7.90, 15.70) | 10.80 (7.90, 14.90) | 11.60 (7.70, 17.20) | 0.001 |
| hemoglobin(g/dL) | 10.88 (2.48) | 11.52 (2.34) | 9.67 (2.29) | <0.001 |
| platelets(10^9/L) | 204.00 (149.00, 277.00) | 208.00 (159.00, 272.00) | 198.00 (127.00, 292.00) | <0.001 |
| RDW(%) | 15.15 (14.00, 16.98) | 14.50 (13.60, 15.60) | 17.20 (15.60, 19.20) | <0.001 |
| lymphocytes(10^9/L) | 1.03 (0.63, 1.60) | 1.08 (0.67, 1.65) | 0.95 (0.58, 1.50) | <0.001 |
| neutrophils(10^9/L) | 8.80 (5.87, 13.43) | 8.59 (5.87, 12.57) | 9.35 (5.86, 14.91) | <0.001 |
| PNI | 38.75 (33.60, 43.80) | 41.44 (37.61, 45.82) | 32.24 (28.25, 36.60) | <0.001 |
| total protein(g/dL) | 5.58 (1.04) | 5.86 (0.95) | 5.07 (1.02) | <0.001 |
| albumin(g/dL) | 3.26 (0.62) | 3.57 (0.43) | 2.69 (0.51) | <0.001 |
| ALT(U/L) | 26.00 (16.00, 54.00) | 27.00 (17.00, 54.00) | 25.00 (15.00, 54.00) | 0.004 |
| AST(U/L) | 39.00 (24.00, 84.00) | 39.00 (24.00, 82.00) | 40.00 (24.00, 86.50) | 0.678 |
| ALP(U/L) | 89.00 (66.00, 128.00) | 85.00 (65.00, 118.00) | 98.00 (69.00, 150.00) | <0.001 |

| | | | | |
|---|---|---|---|---|
| total bilirubin(mg/dl) | 0.70 (0.40, 1.20) | 0.60 (0.40, 1.00) | 0.70 (0.40, 1.50) | <0.001 |
| BUN(mg/dl) | 30.00 (19.00, 48.00) | 27.00 (19.00, 44.00) | 35.00 (21.00, 56.00) | <0.001 |
| creatinine(mg/dl) | 1.40 (0.90, 2.20) | 1.30 (0.90, 2.00) | 1.50 (1.00, 2.50) | <0.001 |
| albuminuria(mg/dl) | 77.00 (30.00, 77.00) | 77.00 (30.00, 77.00) | 77.00 (30.00, 100.00) | 0.978 |
| glucose(mg/dl) | 136.00 (108.00, 184.00) | 146.00 (115.00, 197.00) | 139.00 (113.00, 189.00) | <0.001 |
| LVEF(%) | 46.44 (47.15) | 46.39 (57.41) | 46.53 (15.09) | 0.903 |
| inotropes/vasopressors, n(%) | 1797 (39.9%) | 1030 (35.1%) | 767 (48.7%) | <0.001 |
| CRRT, n(%) | 342 (7.6%) | 177 (6.0%) | 165 (10.5%) | <0.001 |
| ventilation, n(%) | 1801 (40.0%) | 1106 (37.7%) | 695 (44.1%) | <0.001 |
| SOFA | 6.60 (4.00) | 5.94 (3.70) | 7.84 (4.23) | <0.001 |
| APACHE II | 67.15 (24.64) | 62.43 (22.36) | 75.91 (26.23) | <0.001 |
| mortality, n(%) | 1944 (43.1%) | 1036 (35.3%) | 908 (57.7%) | <0.001 |

COPD, chronic obstructive pulmonary disease; CKD, chronic kidney disease; SBP, systolic blood pressure; SpO2, saturation of peripheral oxygen; WBC, white blood cell count; RDW, red cell distribution width; PNI, prognostic nutritional index; ALT, alanine aminotransferase; AST, aspartate aminotransferase; ALP, alkaline phosphatase; BUN, blood urea nitrogen; LVEF, left ventricular ejection fraction; CRRT, continuous renal replacement therapy; SOFA, sequential organ failure assessment; APACHE II, Acute Physiology and Chronic Health Evaluation II.

**Supplementary table 3**. Threshold effect analysis of the relationship between red cell distribution width - albumin ratio and all-cause mortality.

| | Threshold of RAR | HR 95CI% | *p* value |
|---|---|---|---|
| 1-line cox regression model | - | 1.20 (1.15~1.25) | < 0.001 |
| 2-line cox regression model | < 5.25 | 1.64 (1.47~1.84) | < 0.001 |
| | ≥5.25 | 1.19 (1.14~1.24) | < 0.001 |
| Likelihood Ratio test | - | | < 0.001 |

Data were adjusted for age, gender, diabetes, hypertension, CKD, sepsis, PNI score, neutrophils, hemoglobin, albumin, total bilirubin, BUN, creatinine, inotropes or vasopressors, LVEF, CRRT, ventilation, SOFA and APACHE II.

**Supplementary table 4**. Concordance index for five models in predicting the all-cause mortality.

| models | concordance index | *p* value |
|---|---|---|
| RAR | 0.622 (0.609~0.635) | - |
| SOFA | 0.636 (0.623~0.649) | <0.001 |
| SOFA + RAR | 0.658 (0.650~0.676) | |
| APACHE II | 0.682 (0.669~0.695) | <0.001 |
| APACHE II + RAR | 0.695 (0.682~0.708) | |

**Supplementary table 5**. Modified Poisson regression models for the association between red cell distribution width - albumin ratio and all-cause mortality in 1 year.

| RAR | Case/Total | Model 0 | Model 1 | Model 2 | Model 3 |
|---|---|---|---|---|---|
| Quartiles | - | Relative Risk | | | |
| Q1 | 1126/4506 | reference | reference | reference | reference |
| Q2 | 1127/4506 | 1.23 (1.09,1.39) | 1.15 (1.02,1.29) | 1.12 (0.99,1.27) | 1.14 (1.01,1.28) |

| | | | | | |
|---|---|---|---|---|---|
| Q3 | 1126/4506 | 1.74 (1.56,1.94) | 1.61 (1.45,1.79) | 1.53 (1.35,1.73) | 1.54 (1.36,1.74) |
| Q4 | 1127/4506 | 2.14 (1.93,2.38) | 1.98 (1.78,2.19) | 1.85 (1.59,2.16) | 1.83 (1.57,2.13) |
| *p* for trend | - | < 0.001 | < 0.001 | < 0.001 | < 0.001 |
| Per quartile increase | - | 1.30(1.26,1.34) | 1.27 (1.23,1.31) | 1.25 (1.19,1.31) | 1.24 (1.18,1.30) |
| Per unit increase | - | 1.15 (1.14,1.17) | 1.15 (1.13,1.16) | 1.14 (1.11,1.17) | 1.12 (1.09,1.15) |

Model 0: red cell distribution width - albumin ratio without adjust; Model 1: age, gender, diabetes, hypertension, CKD, sepsis, and PNI score were adjusted; Model 2: the variables in Model 1 plus neutrophils, hemoglobin, albumin, total bilirubin, BUN, creatinine, inotropes or vasopressors, CRRT and ventilation were adjusted; Model 3: the variables in Model 2 plus SOFA and APACHE II were adjusted.
